# Climate implications on forest above- and belowground carbon allocation patterns along a tropical elevation gradient on Mt. Kilimanjaro (Tanzania)

**DOI:** 10.1007/s00442-021-04860-8

**Published:** 2021-02-25

**Authors:** Natalia Sierra Cornejo, Christoph Leuschner, Joscha N. Becker, Andreas Hemp, David Schellenberger Costa, Dietrich Hertel

**Affiliations:** 1 Department of Plant Ecology and Ecosystems Research, Albrecht Von Haller Institute for Plant Sciences, Georg August University of Goettingen, Göttingen, Germany; 2 Institute of Soil Science, Center for Earth System Research and Sustainability (CEN), University of Hamburg, Hamburg, Germany; 3 Department of Plant Physiology, Bayreuth University, Bayreuth, Germany; 4 Institute for Ecology and Evolution, Friedrich Schiller University of Jena, Jena, Germany

**Keywords:** Africa, Carbon cycle, Fine roots, Net primary production, Tropical montane forest

## Abstract

Tropical forests represent the largest store of terrestrial biomass carbon (C) on earth and contribute over-proportionally to global terrestrial net primary productivity (NPP). How climate change is affecting NPP and C allocation to tree components in forests is not well understood. This is true for tropical forests, but particularly for African tropical forests. Studying forest ecosystems along elevation and related temperature and moisture gradients is one possible approach to address this question. However, the inclusion of belowground productivity data in such studies is scarce. On Mt. Kilimanjaro (Tanzania), we studied aboveground (wood increment, litter fall) and belowground (fine and coarse root) NPP along three elevation transects (c. 1800–3900 m a.s.l.) across four tropical montane forest types to derive C allocation to the major tree components. Total NPP declined continuously with elevation from 8.5 to 2.8 Mg C ha^−1^ year^−1^ due to significant decline in aboveground NPP, while fine root productivity (sequential coring approach) remained unvaried with around 2 Mg C ha^−1^ year^−1^, indicating a marked shift in C allocation to belowground components with elevation. The C and N fluxes to the soil via root litter were far more important than leaf litter inputs in the subalpine *Erica* forest. Thus, the shift of C allocation to belowground organs with elevation at Mt. Kilimanjaro and other tropical forests suggests increasing nitrogen limitation of aboveground tree growth at higher elevations. Our results show that studying fine root productivity is crucial to understand climate effects on the carbon cycle in tropical forests.

**Supplementary Information:**

The online version contains supplementary material available at 10.1007/s00442-021-04860-8.

## Introduction

Tropical forests cover only 13% of the earth’s land surface but may account for one-third of terrestrial net primary productivity (NPP) (Del Grosso 2008) and store 40–50% of the terrestrial biomass carbon (Houghton 2005; Lewis et al. 2009). Climate change has the potential to reduce the forests’ sink strength for carbon (C) in a hotter and drier climate through reduced CO_2_ uptake, increased respiratory CO_2_ release, or both (Lloyd and Farquhar 2008; Cernusak et al. 2013; Mitchard 2018). Changing temperature and moisture conditions may also affect the patterns of carbohydrate allocation to aboveground and belowground sinks, i.e. wood increment, the production of leaves, flowers and fruits, root production, root exudation, and C transfer to mycorrhiza. For example, warmer temperatures tend to accelerate soil nitrogen (N) cycling with the consequence of better nutrient supply and higher aboveground productivity at the cost of root productivity (Robertson and Groffman 2015; Moser et al. 2011). Yet, higher temperatures also increase the atmospheric vapor pressure deficit, which can induce drought stress with negative effects on forest productivity even in situations when soil moisture is not limiting (Yuan et al. 2019). On the other hand, climate warming could also lead to increased precipitation, as is predicted by climate change scenarios for parts of East Africa (Niang et al. 2014), possibly hampering root growth in already wet soils. Thus, wood production and timber yield may decrease or increase with climate change as a consequence of allocation shifts, independently of changes in total forest productivity. How long-term change toward a hotter climate with higher evaporative demand influences the C cycle of tropical forests is hence only poorly understood, further studies being needed (Wood et al. 2012). One reason for this lack of understanding is that most productivity studies in tropical forests have considered only aboveground components of productivity (wood and leaf production) while ignoring belowground productivity. Measuring root productivity is labor-intensive, especially with respect to the fine root fraction (conventionally roots < 2 mm in diameter). The synchronization of root growth and root mortality, together with the high spatial heterogeneity of the root system makes the observation of root dynamics notoriously difficult (Eissenstat et al. 2000). Consequently, various authors have used estimates of root production to obtain total (above- and belowground) productivity, but these production figures should be considered with caution.

A second reason for this limited understanding is the existence of contradicting evidence with respect to the temperature dependence of net primary productivity (NPP) of tropical and subtropical forests, i.e. the balance of photosynthetic carbon gain and carbon loss through autotrophic respiration. The climatic control of forest NPP is still a matter of debate, as several studies provide evidence for a direct temperature dependence of productivity (Huxman et al. 2004; Leuschner et al. 2013; Lichstein et al. 2014; Chu et al. 2015), while other investigations point to a more prominent role of solar radiation, tree functional traits and stand structural attributes as principal drivers of forest NPP (Luyssaert et al. 2007; Michaletz et al. 2014; Fyllas et al. 2017).

Some insights on the importance of climatic drivers can be gained from the comparative study of forest productivity along climate or elevation gradients. Even though these gradients are often linked with a change in other environmental factors, they have the advantage of informing long-term acclimative and adaptive responses of trees to climate warming. We are aware of only two comprehensive studies along tropical elevation gradients that covered the major above- and belowground components of forest productivity, i.e. the study by Girardin et al. (2010) and Malhi et al. (2017) in the Peruvian Andes, and the study by Moser et al. (2011) and Leuschner et al. (2013) in the Ecuadorian Andes. Since root production was also investigated, these two pioneer studies allow conclusions on carbon allocation shifts along the elevation gradient that may relate to alterations in temperature, moisture and nutrient availability. Both studies found increased carbon allocation to root production with increasing elevation, but the extent to which the shift occurred, differed. Moreover, both studies report deviating elevational trends for total NPP, carbon partitioning and fine root carbon residence time, highlighting the regional character of the observed patterns. This suggests that local differences in climate and geology, and probably also in the biogeographical setting, may lead to deviating patterns of NPP and carbon allocation change with elevation in different tropical mountains (Malhi et al. 2011). Comprehensive studies of forest NPP and its components along elevational gradients are lacking in the African and Asian tropical forest realms so far.

Measuring aboveground and belowground productivity in combination with the corresponding biomass C pools allows the estimation of ecosystem carbon cycling rates and to distinguish ‘fast’ and ‘slow’ C pools, i.e. plant components with low or high carbon residence time (CRT). Comparative analyses of CRT in different ecosystem types along climate or elevation gradients may deepen our understanding of abiotic and biotic controls of ecosystem C cycling (Girardin et al. 2010; Malhi et al. 2017).

The moist tropical lowland forests of Africa are on average less species-rich than neotropical forests, but mean aboveground biomass is higher and average tree height greater (Hemp et al. 2017; Sullivan et al. 2017). In fact, biomass in tropical montane forests on Mt. Kilimanjaro is higher than in Andean montane forests (Ensslin et al. 2015). However, it is unknown if this higher biomass is also reflected in a higher productivity. Less is known about the carbon cycle of African tropical forests compared to their American and Asian counterparts. Apart from very few NPP measurements in lowland forest plots (e.g. Moore et al. 2018), only one productivity assessment has been done in an African tropical montane forest (Rwanda; Nyirambangutse et al. 2017). Mount Kilimanjaro, in northern Tanzania, with a forest belt covering an elevation gradient of approx. 2000 m. from lower montane forest to the subalpine *Erica* forest, the highest forests of Africa (Hemp 2005), offers unique opportunities to study the variation in forest NPP and carbon allocation with temperature and moisture change, as soils are of similar origin (volcanic) and age. By determining all major components of NPP (aboveground wood increment, aboveground litter fall, fine root and coarse root production) in four characteristic forest types, total NPP and aboveground/belowground carbon allocation patterns were derived and related to temperature, rainfall and soil chemistry patterns on the mountain. We further calculated nitrogen use efficiency of productivity (NUE), carbon residence time, and the transfers of C and N with aboveground and belowground (root) litter fall to the soil for the different forest ecosystems in order to characterize putative factors influencing productivity along the elevation gradient.

The following hypotheses guided our research: (1) Total NPP declines and C allocation shifts to the fine root system as a consequence of increasing N limiting conditions with elevation; (2) nutrient use efficiency of productivity is higher at high elevations as a response to low soil N availability; (3) C and N fluxes from fine root mortality to the soil are more important than from leaf litter at high elevations as a result of plant species adaptation to the harsh conditions.

## Material and methods

### Study site

The study was conducted on the southern and southeastern slopes of Mount Kilimanjaro, northern Tanzania (3°4´33´´S, 37°21´12´´E), in the framework of the KiLi project, an interdisciplinary research project on biodiversity, biotic interactions and biogeochemical cycles in the main ecosystems of the mountain. From the foothills to the mountain top, a characteristic sequence of vegetation belts from savanna woodland through tropical montane forest to alpine heathland is found (Hemp 2006). The present study investigates four different types of tropical montane forest inside Kilimanjaro National Park at elevations from 1800 to 3910 m a.s.l. Along three elevation transects, twelve study plots of 0.25 ha were demarcated in the four forest types, resulting in threefold replication at the plot level (Table 1). Detailed information about the forest ecosystems and their plant species composition is given in Hemp (2006). The lower montane forest (1800–2000 m a.s.l.) is characterized by *Macaranga kilimandscharica*, *Agauria salicifolia* and, to a lesser degree, *Ocotea usambarensis*. The middle montane forest (*Ocotea* forest, 2100–2800 m a.s.l.) is dominated by *Ocotea usambarensis, Ilex mitis, Xymalos monospora* and the tree fern *Cyathea manniana,* and contains a dense understory layer. The upper montane forest (*Podocarpus* forest, 2800–3000 m a.s.l.) hosts *Podocarpus latifolius* as the dominant tree species together with *Hagenia abyssinica* and *Prunus africana*. In the subalpine belt (i.e. the treeline ecotone sensu Körner (2012)) (3500–3900 m a.s.l), *Erica* bushlands with some remnants of *Erica trimera* forests are dominant, which form the tree line.

**Table 1:** Some physiographic and stand structural characteristics of the plots studied in the four forest types on Mt. Kilimanjaro. Given are means ± SE (*n* = 3)

	Lower montane forest	*Ocotea* forest	*Podocarpus* forest	*Erica* forest
Elevation (m a.s.l.)	1800–2040	2120–2750	2800–2970	3500–3910
MAT (°C)^a^	14.7 ± 0.3	11.3 ± 0.7	9.3 ± 0.3	6.0 ± 1.2
MAP (mm)^b^	2227 ± 215	1995 ± 285	1545 ± 178	1051 ± 75
C:N (topsoil)^c^	15.9 ± 0.7	19.3 ± 1.0	18.6 ± 1.3	19.6 ± 0.7
pH (KCl) (topsoil)^c^	4.23–4.34	3.49–4.23	3.83–5.35	4.45–4.54
WFPS (%)^d^	21.6 ± 8.4	41.1 ± 13.2	36.1 ± 11.5	n.d
AGB (Mg ha^−1^)^e^	267.4 ± 45.0	328.8 ± 83.6	404.4 ± 49.7	32.5 ± 7.2
Stem density (n ha^−1^)^e^	397 ± 39	311 ± 36	523 ± 33	2091 ± 1196
Basal area (m^2^ ha^−1^)^e^	47.1 ± 9.8	46.1 ± 7.7	55.7 ± 5.1	14.9 ± 3.3
Mean tree height (m)^e^	15.6 ± 0.9	16.0 ± 1.8	15.5 ± 1.3	4.9 ± 0.1
LAI (m^2^ m^−2^)^f^	6.25 ± 0.03	5.43 ± 1.16	5.09 ± (0.54)	2.15 ± 0.22
Leaf C:N ratio (mg mg^−1^)	19.2 ± 0.6	21.2 ± 1.3	29.4 ± 3.2	37.9 ± 0.8

^a^MAT: mean annual temperature [Appelhans et al. (2015)]

^b^MAP: mean annual precipitation [Hemp (2006)]

^c^Soil C:N and pH range values (0–10 cm of the topsoil) (Becker, unpublished data)

^d^WPFS water-filled pore space (0–5 cm topsoil) (weighted mean of measurements in the long and short rainy seasons, and the long and short dry seasons) [Gütlein et al. (2018)]

^e^AGB: aboveground biomass stand structural data of trees > 10 cm DBH [Hemp (unpublished data)], in case of Erica forest DBH ≥ 5 cm (Schellenberger Costa, unpublished data)

^f^LAI: leaf area index [Rutten et al. (2015)]

Mean annual temperature ranges from 15 °C in the lower montane forest to 4 °C in the highest *Erica* forest (Appelhans et al. 2015). Rainfall exhibits a bimodal seasonal distribution on Mt. Kilimanjaro with a long rainy season from March to May and a short rainy season around November (Hemp 2006). Mean annual precipitation decreases from 2200 mm in the lower montane forest (1800 m a.s.l.) to 1000 mm in the subalpine *Erica* forest belt (3900 m a.s.l.) (Hemp 2006).

The soils on the southern slopes of Mt. Kilimanjaro developed from the same volcanic deposits and are thus of similar age (Dawson 1992). In the four forest types, andosols with folic, hystic or umbric properties are predominant, indicating high topsoil carbon contents (Zech 2006).

### Determination of NPP

In all 12 plots, we measured aboveground wood increment, aboveground litterfall production, coarse root production and fine root production as the main components of net primary productivity over a 24-month interval (Aug 2014–Jul 2016) (Clark et al. 2001a). In the case of fine root production, the measuring period covered only one year (Aug 2015–Jul 2016). We assumed that a steady-state in the production of leaves, twigs, inflorescences and fruits, i.e. equality between the production of new organs and litterfall existed in the canopy (Aragão et al. 2009), as well as in the fine root system (Graefe et al. 2008).

#### Aboveground productivity and coarse root production

The production of aboveground biomass was derived from stem increment measurements with dendrometer tapes (UMS, München, Germany) mounted permanently on 40 stems per plot (480 stems in total). In the 0.25 ha-plots, the 40 stems were selected from the more abundant tree species and from different diameter classes to include the whole spectrum of stem diameters above 10 cm DBH. In case of the *Erica* forest, the DBH threshold was set to ≥ 5 cm, as the 5–10 cm-class contributes significantly to total coarse wood biomass in this ecosystem. Dendrometer tapes were placed at 1.3 m height above ground. When buttresses or stem anomalies were present, we moved the dendrometers some centimeters higher or lower. Tape readings were conducted at monthly intervals from August 2014 to July 2016. Aboveground biomass production was calculated as the difference in biomass between the first and last reading, divided by interval length. To obtain aboveground biomass from DBH and tree height, we applied the pantropical allometric equation of Chave et al. (2014) (Eq. 1):

1 $$AGB = 0.0673\left( {\rho D^{2} H} \right)^{0.0976}$$

in which AGB is the aboveground biomass estimate (in kg per tree), ρ is the specific gravity of the wood (dry weight per fresh volume in kg m^−3^), D the trunk diameter (DBH in cm), and H tree height (in m). AGB covers coarse wood biomass, and twig and leaf mass. This NPP component is termed hereafter NPP-aboveground wood. The procedures to measure specific wood gravity (wood density) and tree height in the plots are described in Ensslin et al. (2015) and Schellengerger Costa et al. (2017). When wood density data were not available from the plots (in 2 species and 16 stems), values were taken from the global wood density database (Zanne et al. 2009). For stems that were not equipped with dendrometer tapes in the plots, we used increment rates averaged per species and per plot. For tree species that were not monitored, we used increment rates averaged over all measured species in the plot.

The production of coarse roots, i.e. roots with diameter > 5 mm, was derived from measured aboveground biomass increment applying the equation given for tropical forests by Cairns et al. (1997) (Eq. 2).

2 $$BGB = \exp \left( { - 1.0587 + 0.8836\ln \left( {AGB} \right)} \right)$$

with BGB being the belowground biomass and AGB the aboveground biomass, both in Mg ha^−1^. The equation was primarily derived for root diameters > 5 mm, but some of the included studies considered also fine roots. Since fine roots contribute with only a few percent to belowground biomass, we used the measured AGB difference between first and last reading for estimating the production of coarse root biomass (hereafter: NPP-coarse roots), the main component of belowground biomass.

To express NPP in terms of carbon (C), we used a C content of the biomass of 48.2% for all NPP components (Thomas and Martin 2012). Carbon residence time (CRT) of the NPP components was calculated by dividing the amount of C contained in the biomass by the C accumulated by the formation of new biomass (productivity) (Malhi et al. 2004). This was done for all NPP components except for aboveground litterfall, as canopy biomass was not measured.

#### Aboveground litter production and its components

Aboveground litterfall was measured with ten randomly placed litterfall traps (size: 1 m × 1 m) that were collected at monthly intervals from August 2014 to July 2016. PVC tubes were inserted at the corners of the traps and a nylon mesh of 1 mm mesh width placed 20 cm above ground between the four tubes. In the *Ocotea* forest, the net was located at 80 cm height to collect the litter above the understory vegetation layer. In the laboratory, the collected material was separated for leaves, twigs (diameter < 20 mm) and other litter components (inflorescences, fruits, leaf fragments, unidentified material), oven-dried at 60 °C for one week, weighed and the mass expressed as litterfall per time interval and ha^−1^ (hereafter: NPP-aboveground litterfall). To obtain total aboveground NPP (NPP-aboveground), the NPP-aboveground litterfall was added to the NPP-aboveground wood.

The C and N content of all litter fractions was measured through gas chromatography with a CN elemental analyzer (Vario EL III, Hanau, Germany). We analyzed five samples per plot, each consisting of the mix of two samples from two sampling dates (May 2016 for the long wet season and July 2016 for the long dry season). For direct comparison with the fine root litter fraction, we calculated the leaf litter C:N ratio and the annual transfer of C and N with leaf litterfall to the soil, using plot-level means. The nitrogen use efficiency (NUE) of productivity was calculated in the different forest types after Vitousek (1982) by dividing annual aboveground litter mass by litter N content.

#### Fine root production

The available methods for measuring fine root productivity (NPP-fine roots) have reported divergent results in several studies (Hertel and Leuschner 2002; Hendricks et al. 2006; Moser et al. 2010). Therefore, it is advisable to combine at least two approaches to get an impression of the possible bias in the data (Clark et al. 2001a). In this study, we applied the sequential coring approach (Persson 1980; Majdi 1996) together with the ingrowth core method (Majdi et al. 1996). The first method has been widely used but is labor-intensive and may be problematic especially at sites with low root mass seasonality (Vogt et al. 1998; Hertel and Leuschner 2002). The second method has been found to underestimate fine root production, but it can be conducted with a relatively high number of replicates and may serve for comparing root production at different sites when root growth is fast, as is typically the case in tropical forests (Vogt et al. 1998). While the absolute productivity values obtained with the ingrowth core method seem to underestimate fine root production and may better be used to characterize the regeneration potential of the fine root system after disturbance, the figures from a large number of locations may nevertheless give an impression of relative differences in plot-level fine root productivity. On the other hand, some authors consider that minirhizotrons might be a quite reliable method to estimate fine root production, as the same roots are followed through time and a direct observation of death and growth is possible (Hendricks et al. 2006; Moser et al. 2010). However, it was not possible to include this approach on our study due to logistic issues.

##### Sequential coring approach

Due to the high labor effort needed to collect and process the samples, the sequential coring approach was conducted in only one of the three elevation transects. On four occasions in the period August 2015–July 2016 (Aug 2015, Jan 2016, May 2016, Jul 2016), 15 soil cores (3.5 cm in diameter) per plot and date were extracted down to 40 cm depth and stored at 5 °C until analysis. Coring on the subsequent date was conducted at a distance of 20 cm to the last one, moving to the corners of a square. In the laboratory, samples were washed through a sieve of 200 μm mesh size and root fragments > 10 mm in length and ≤ 2 mm in diameter were picked out. Living and dead fine roots were distinguished under a stereomicroscope based on the degree of root elasticity, the cohesion of cortex, periderm and stele, and the turgidity of the cortex (Leuschner et al. 2001b). Fine roots from woody plants were selected by means of root morphology and branching patterns and the lack of visible suberinization. Only these roots were considered for further analysis. Root samples were dried at 70 °C for 48 h and weighed. Additionally, we applied the protocol proposed by Van Praag et al. (1988) and modified by Hertel and Leuschner (2002) to cover necromass fragments < 10 mm in length, which represent a large part of the necromass. Six samples per plot were selected, the larger root fragments (˃ 10 mm) extracted by hand, and the remaining of the sample spread homogenously on a filter paper (730 cm^2^) subdivided into 36 squares. In six randomly chosen squares all root fragments (mostly necromass) were extracted under the stereomicroscope and the mass determined by drying and weighing. This small necromass fraction was multiplied by six to extrapolate to the entire sample and added to the necromass fraction > 10 mm in length to obtain an estimate of total fine root necromass. We then extrapolated the mass of the small-root fraction to the fine root necromass of the remaining samples that were not included in this more detailed analysis, using linear regression equations between the masses of the small-root fragments and the larger dead fine root fraction. A mean ratio of small to large root fractions was used in cases when a regression equation could not be applied.

Fine root production was calculated with the “minimum–maximum” approach (Edwards and Harris 1977; McClaugherty et al. 1982) by subtracting the lowest mean fine root mass (biomass plus necromass; *n* = 15 cores) from the highest mean in the 1-year measuring period, considering only significant differences between dates (Vogt et al. 1986). As this method does not take into account the simultaneous occurrence of fine root production and root mortality in the study period, underestimation of fine root production is possible (McClaugherty et al. 1982). For the other two transects, where the sequential coring method was not conducted, fine root production was estimated from the fine root biomass data of these transects (Sierra Cornejo et al. 2020) using a regression between root biomass and root production determined in the first transect. NPP-fine roots and NPP-coarse root were added to obtain NPP-belowground.

##### Ingrowth core study

As a second independent method, an ingrowth core study was conducted in the 12 plots of the three transects. In September 2014 and February 2015 (dry season), ten ingrowth cores (0–40 cm soil depth) were placed at random locations in each plot. After soil extraction with a 3.5 cm-soil corer, all visible roots were removed in situ by hand and the original hole was refilled with the root-free soil. The original soil layer sequence and bulk density were restored as well as possible. The location of the core was precisely marked with three thin plastic sticks and a PVC tube on the surface of the same diameter of the core. No mesh was inserted in the soil to avoid growth barriers and further disturbance of the soil texture (Hertel et al. 2013; Kubisch et al. 2017). The locations were resampled after one year with the same corer. Samples were processed in the laboratory following the same protocol as used in the sequential coring approach but without the detailed study on the very small root particles. This step was not necessary because the retrieved cores contained mostly newly ingrown living fine roots. Fine root production was calculated as ingrown fine root mass (living and dead roots) in relation to the length of the period between the start of recolonization and harvest (Vogt et al. 1998). To determine the recolonization starting point in the four forest types, we installed four additional ingrowth cores per plot and resampled them at monthly intervals during the subsequent four months. According to this side study, fine roots started to grow into the cores in the lower montane forest two months after exposure, and in the *Ocotea, Podocarpus* and *Erica* forests after three months. These periods were subtracted from the 1-year-long exposure period. Fine root production was expressed at an annual basis (in Mg ha^−1^ year^−1^). Due to logistic problems, we could not retrieve the ingrowth cores from four of the 12 plots (one plot per forest type). The missing production values were estimated from the mean ratio between fine root production and fine root biomass of a forest type.

Analagous to aboveground nutrient use efficiency, we calculated the nitrogen use efficiency of fine root production for the four forest types by dividing fine root production by the N content of fine root biomass. Fine root growth is set equal with fine root litter production, assuming a steady state between fine root production and mortality. This assumption is supported by the results of a study with minirhizotrons along an elevation gradient in the Ecuadorian Andes, where similar fine root growth and mortality rates were found (Graefe et al. 2008). In addition, it has been shown that a major trigger of fine root production is the response to fine root death by compensatory fine root growth (Leuschner et al. 2001a; Kubisch et al. 2017), fine root production depending on factors determining fine root lifespan (Eissenstat and Yanai 1997; Hertel and Leuschner 2002; Hertel et al. 2013). For this calculation, we used the fine root N content data of the fine root biomass survey by Sierra Cornejo et al. (2020).

Carbon and nitrogen fluxes to the soil with root mortality were estimated for the four forest types by multiplying the C and N content of the fine root biomass (Table S1, Sierra Cornejo et al. 2020) with the calculated fine root productivity in the plot, assuming equivalence of productivity and mortality.

### Statistical analysis

All statistical analyses were conducted in R-3.4.0 (R Core Team 2017). The influence of forest type on total NPP was analyzed with an ANOVA using plot-level means of productivity data. Differences between forest types were detected with a Tukey HSD post-hoc test. Linear and non-linear regressions were calculated to relate (1) the different NPP components, the partitioning of carbon to different sinks, carbon residence time, and nutrient use efficiency (fine root or aboveground production) to elevation, and (2) the NPP components to climatic, edaphic and stand structural variables. Again, plot means were used in the regression analyses. The normality and homoscedasticity of the residuals were visually inspected. We used a significance level of *P* < 0.05 throughout the study.

## Results

### Elevational change in NPP

**Fig. 1:**
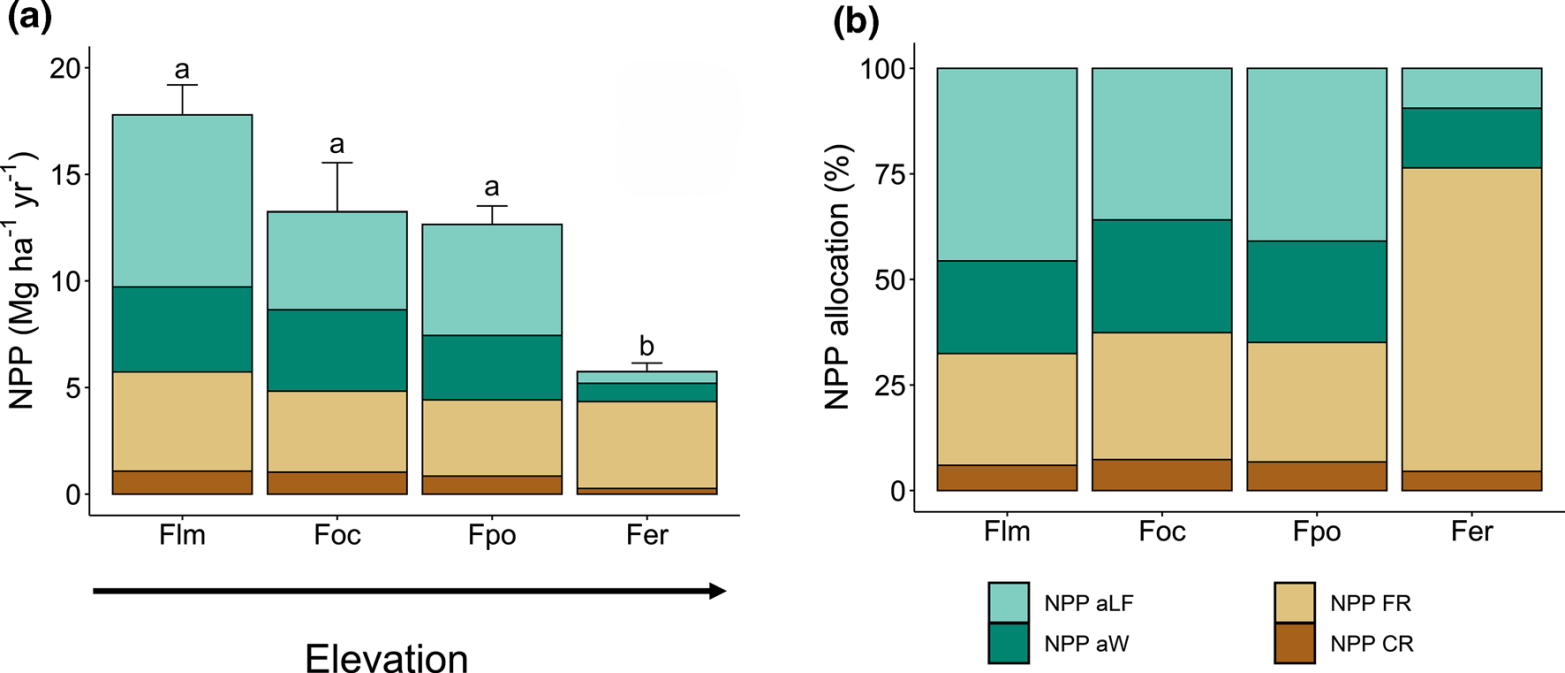
**a** Net primary productivity (NPP) of the four forest types by components, and **b** relative allocation of carbon to the different NPP components. All values are means ± SE (*n* = 3). Different lower case letters indicate significant differences between total NPP values of the different forest types according to a Tukey HSD test (*P* < 0.05). *Flm* lower montane forest, *Foc* *Ocotea* forest, *Fpo* *Podocarpus* forest, *Fer Erica* forest, *aLF* aboveground litterfall, *aW* aboveground wood, *CR* coarse roots, *FR* fine roots

**Fig. 2:**
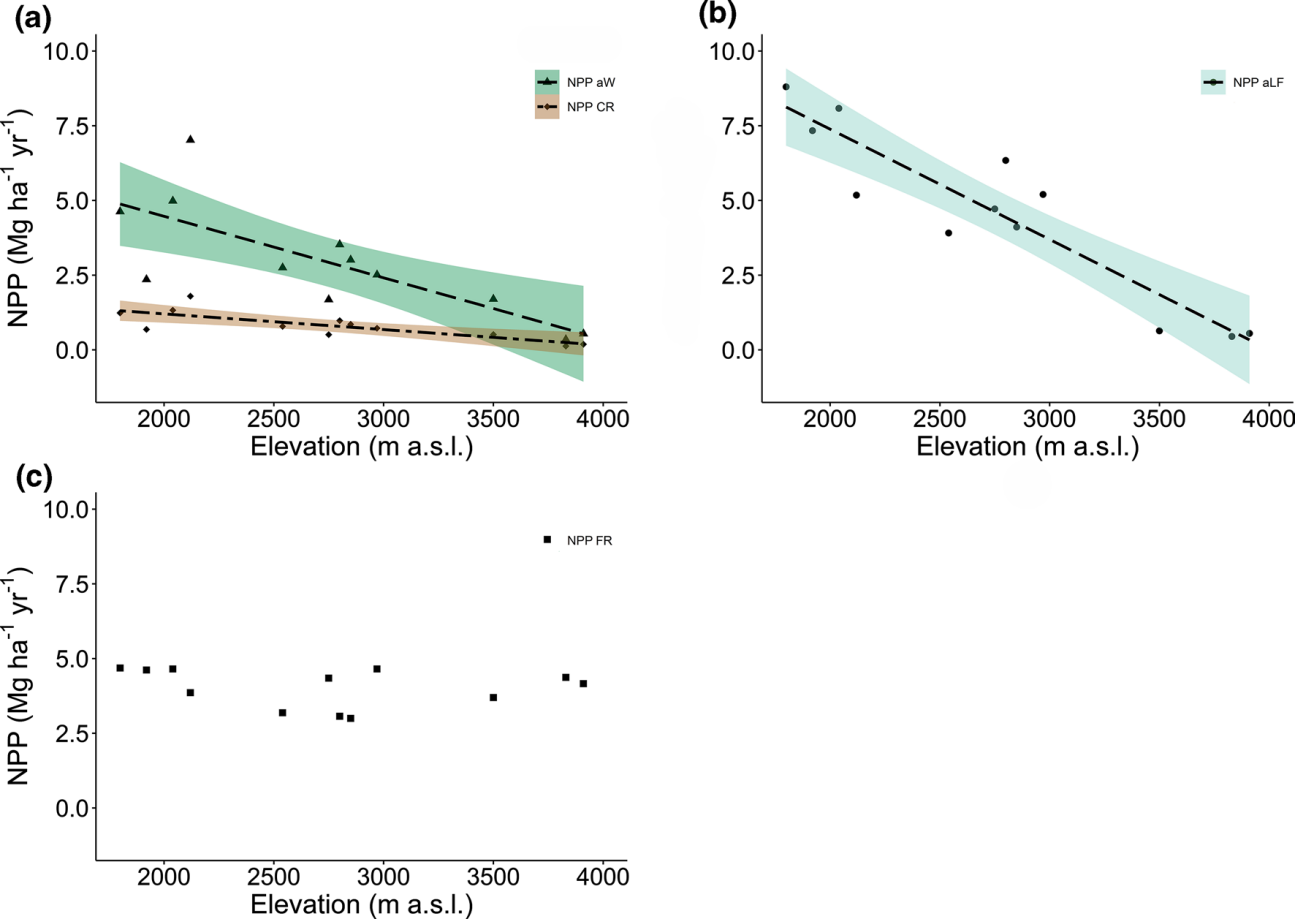
Dependence on elevation of **a** aboveground wood productivity and coarse root productivity, **b** aboveground litter production, and **c** fine root productivity in the four forest types. Dashed lines indicate linear regressions, colored areas the 95% confidence interval (NPPaW: *r*^*2*^ = 0.60; *P* < 0.01; NPPCR: *r*^*2*^ = 0.62, *P* < 0.01; NPPaLF: *r*^*2*^ = 0.85, *P* < 0.001; NPPFR show no change with elevation; in all cases *n* = 12). *aLF* aboveground litter fall, *aW* aboveground wood, *CR* coarse roots, *FR* fine roots

**Fig. 3:**
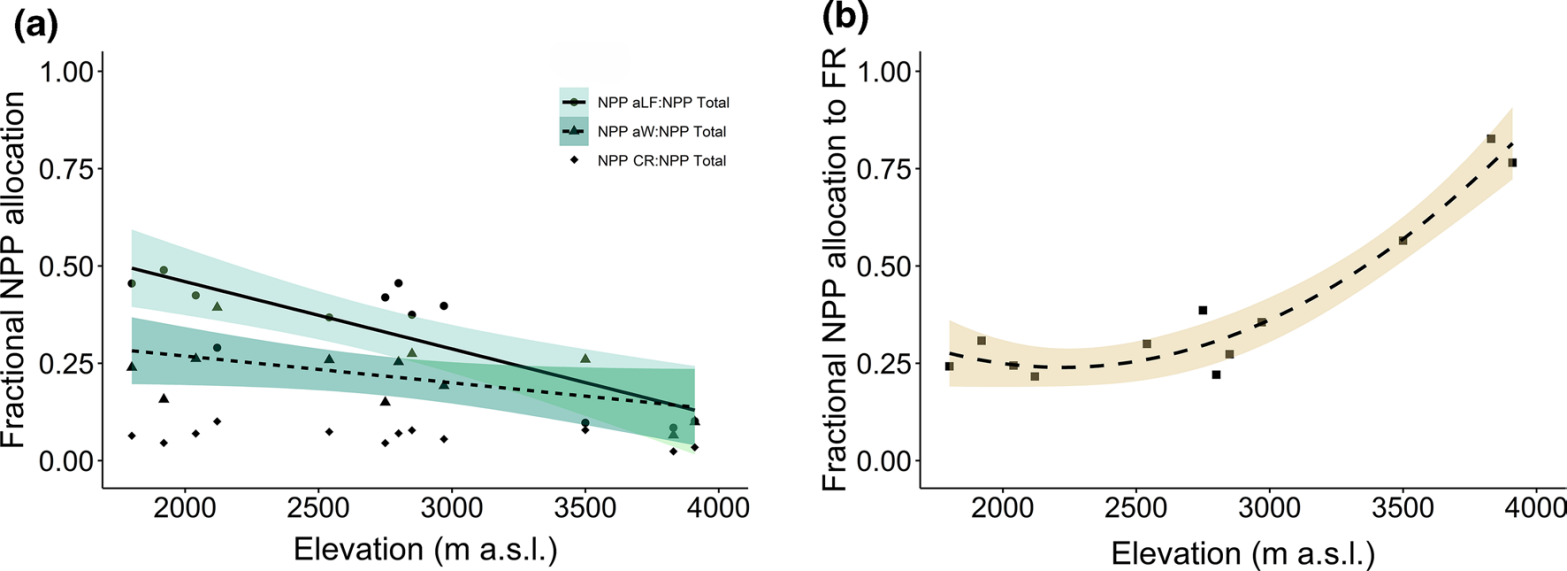
Change with elevation in the proportion of **a** aboveground litter (leaves, fruits, twigs), aboveground wood productivity, and coarse root productivity, and **b** fine root productivity in total NPP in the four forest types. The different straight lines indicate linear regressions, the curved line a 2nd-order polynomial regression, the colored areas the 95% confidence intervals. (NPPaLF: NPPtotal: *r*^*2*^ = 0.68, *P* < 0.01; NPPaW: NPPtotal showed no change with elevation: *r*^*2*^ = 0.31, *P* < 0.1; NPPCR: NPPtotal showed no change with elevation: *r*^*2*^ = 0.23, *P* = 0.12; NPPFR: NPPtotal: *r*^*2*^_*adj*_ = 0.93, *P* < 0.001; in all cases *n* = 12). *aLF* aboveground litter fall, *aW* aboveground wood, *CR* coarse roots, *FR* fine roots

Total NPP (NPP-aboveground plus NPP-belowground) decreased from the lower montane forest at 1800–2040 m a.s.l. (17.8 ± 1.4 Mg ha^−1^ year^−1^) to the subalpine *Erica* forest at 3500–3910 m (5.8 ± 0.4 Mg ha^−1^ year^−1^) (Table 2, Fig. 1). All NPP components except fine root productivity decreased continuously with elevation in size, with the steepest slope detected for NPP-aboveground litterfall (reduction to less than 10%), a moderate slope in NPP-aboveground wood (reduction to about a quarter), and the slightest slope in NPP-coarse roots (Table 2, Fig. 2). The relative proportion of NPP-aboveground litterfall and NPP-aboveground wood changed only little between 1800 and 3200 m to drop sharply toward the subalpine *Erica* forest (Table 2). In contrast, fine root productivity calculated with the sequential coring approach remained relatively constant across the elevation transect with 3.6–4.7 Mg ha^−−1^ year^−1^. In the lower montane, the *Ocotea* and the *Podocarpus* forest, 35–45% of total NPP was contributed by aboveground litter production (mostly leaves, twigs < 20 mm, and fruits), 25–30% by NPP-fine roots, and 20–25% by NPP-aboveground wood. NPP partitioning was different in the *Erica* forest at the highest elevation, where about 70% of total NPP referred to fine root productivity, while aboveground NPP (NPP-wood and NPP-litter fall) contributed only c. 25% (Fig. 1). Consequently, the proportion of NPP-fine roots to total NPP increased greatly at the transition from the *Podocarpus* to the *Erica* forest upslope, indicating a large belowground shift in carbohydrate allocation above 3000 m (Fig. 3). This shift was associated with a dramatic increase in the fine root:leaf litter ratio, as leaf litterfall was very small and fine root productivity relatively high in the *Erica* forest (Table 2).

**Table 2:** Net primary productivity (NPP) and its components, carbon residence time (CRT) in different biomass components, and nitrogen (N) use efficiency of productivity in the four forest types

	Lower montane forest	*Ocotea* forest	*Podocarpus* forest	*Erica* forest
Production (Mg ha^−1^ year^−1^)				
NPP total	17.79 ± 1.41 (8.47)	13.24 ± 2.31 (6.43)	12.65 ± 0.87 (6.16)	5.75 ± 0.40 (2.78)
Aboveground production	12.06 ± 1.19 (5.83)	8.42 ± 1.89 (4.14)	8.23 ± 0.83 (4.04)	1.40 ± 0.47 (0.69)
Canopy litterfall	8.07 ± 0.42 (3.91)	4.60 ± 0.37 (2.30)	5.21 ± 0.64 (2.58)	0.54 ± 0.05 (0.28)
Leaves	6.14 ± 0.39 (2.98)	3.24 ± 0.34 (1.64)	3.18 ± 0.36 (1.61)	0.009 ± 0.003 (0.005)
Branches	0.95 ± 0.19 (0.45)	0.45 ± 0.09 (0.22)	0.59 ± 0.12 (0.29)	0.32 ± 0.05 (0.16)
Rest	0.99 ± 0.04 (0.48)	0.91 ± 0.18 (0.44)	1.44 ± 0.17 (0.69)	0.22 ± 0.01 (0.11)
Wood	3.99 ± 0.82 (1.92)	3.82 ± 1.63 (1.84)	3.02 ± 0.29 (1.45)	0.86 ± 0.42 (0.42)
Belowground production	5.73 ± 0.22 (2.64)	4.83 ± 0.49 (2.29)	4.42 ± 0.48 (2.12)	4.35 ± 0.08 (2.08)
Fine roots Sequential coring	4.65 ± 0.02 (2.12)	3.80 ± 0.34 (1.79)	3.57 ± 0.54 (1.71)	4.07 ± 0.20 (1.95)
Fine roots Ingrowth core	1.14 ± 0.14 (0.51)	1.12 ± 0.31 (0.54)	1.42 ± 0.11 (0.66)	0.47 ± 0.06 (0.22)
Coarse roots	1.08 ± 0.20 (0.52)	1.03 ± 0.39 (0.50)	0.85 ± 0.07 (0.41)	0.27 ± 0.12 (0.13)
% NPP Aboveground litterfall	46 ± 2	36 ± 4	41 ± 2	9 ± 1
% NPP Aboveground wood	22 ± 3	27 ± 7	24 ± 2	14 ± 6
% NPP Fine roots	26 ± 2	30 ± 5	28 ± 4	72 ± 8
% NPP Coarse roots	6 ± 1	7 ± 2	7 ± 1	5 ± 2
CRT Aboveground wood (year)	68.8 ± 5.1	98.9 ± 15.6	133.8 ± 9.4	48.2 ± 10.8
CRT Coarse roots (year)	45.5 ± 2.9	62.4 ± 8.8	81.6 ± 4.9	33.0 ± 6.7
CRT Fine roots (year)	0.52 ± 0.01	0.36 ± 0.04	1.19 ± 0.28	0.64 ± 0.23
NUE (N) Canopy (g g^−1^)	70.2 ± 4.8	72.1 ± 1.7	74.4 ± 7.1	111.9 ± 9.7
NUE (N) Fine roots (g g^−1^)	65.0 ± 12.7	58.9 ± 2.2	65.2 ± 1.2	145.1 ± 6.6
Fine root: leaf litter ratio (g g^−1^)	0.76 ± 0.04	1.19 ± 0.14	1.15 ± 0.19	535.60 ± 124.48

Given are means ± SE (n = 3). Values in parentheses give carbon fluxes (in Mg C ha^−1^ year^−1^)

*NPP-belowground* NPP-fine roots (Sequential coring) plus NPP-coarse roots

Fine root productivity estimated with the ingrowth core method gave three to four times (in case of the *Erica* forest even eight times) smaller annual productivity values than the corresponding sequential coring data (Table S2). As for the sequential coring data, no change with elevation was visible for the NPP-fine roots (IC) data (1.1.–1.4 Mg ha^−1^ year^−1^), but a reduction to the half occurred in the *Erica* forest (Table 2).

### Environmental drivers of NPP

Total NPP and its components aboveground litter, aboveground wood and coarse root productivity showed a tight positive relation to mean annual temperature (and negative one to elevation; Table 3). The positive relation to MAP was also tight and in NPP-aboveground wood and NPP-coarse roots even closer than the relation to temperature. Total NPP and NPP-aboveground litterfall were negatively related to soil C:N ratio, which was not the case in the other NPP components. In contrast, fine root productivity was related neither to elevation, climatic or soil factors nor to aboveground biomass or basal area (Table 3). Some of the biotic and abiotic factors were strongly correlated (Table S3).

**Table 3:** Regression analysis relating the different productivity components to physiographic, edaphic and climatic, and stand structural variables across the four forest types on Mt. Kilimanjaro

	Elevation		Mean annual temperature		Mean annual precipitation		Soil C:N ratio		pH (KCl)		Aboveground biomass		Basal area	
	*r*^*2*^	*P*	*r*^*2*^	*P*	*r*^*2*^	*P*	*r*^*2*^	*P*	*r*^*2*^	*P*	*r*^*2*^*/ r*^*2*^*adj*	*P*	*r*^*2*^	*P*
NPP total	− **0.87**	** < 0.001**	**0.76**	** < 0.001**	**0.61**	** < 0.01**	− **0.43**	** < 0.05**	− 0.09	n.s	**0.47**	** < 0.05**	**0.54**	** < 0.01**
NPP aLF	− **0.85**	** < 0.001**	**0.79**	** < 0.001**	**0.53**	** < 0.01**	− **0.46**	** < 0.05**	− 0.01	< 0.1	**0.68**	< 0.01*	**0.47**	** < 0.05**
NPP aW	− **0.60**	** < 0.01**	**0.46**	** < 0.05**	**0.55**	** < 0.01**	− 0.21	n.s	− 0.21	n.s	**0.32**	** < 0.01**	**0.60**	** < 0.01**
NPP CR	− **0.62**	** < 0.01**	**0.48**	** < 0.05**	**0.55**	** < 0.01**	− 0.21	n.s	− 0.20	n.s	**0.64**	** < 0.01**	**0.63**	** < 0.01**
NPP FR	− 0.04	n.s	0.05	n.s	0.01	n.s	− 0.09	n.s	− 0.01	n.s	-0.18	n.s	-0.11	n.s

NPP in Mg ha^−1^ year^−1^, elevation in m a.s.l., mean annual temperature in °C, mean annual precipitation in mm, aboveground biomass in Mg ha^−1^ and basal area in m^2^ ha^−1^. Significant relations are marked in bold (*P* < 0.05); nonlinear relations are indicated by (*) and the r^2^_*adj*_ is given. Negative relations are indicated by (−)

Given is the *r*^2^ (adjusted *r*^2^ in the case of nonlinear relations) and the *P* value of the relationships

*aLF* aboveground litter fall, *aW* aboveground wood, *CR* coarse roots, *FR* fine roots

### Carbon residence time and nitrogen use efficiency

Carbon residence time (CRT) as the quotient of productivity and biomass in an NPP component ranged between 48 and 134 years in the aboveground biomass (wood) fraction, and between 33 and 82 years in the coarse root fraction (Table 2). CRT was highest in the conifer-dominated *Podocarpus* forest, and lowest in the *Erica* forest, resulting in a humped-shaped relation with elevation (*r*^*2*^*adj* = 0.80, *P* ˂ 0.001) (Fig. S1). We determined CRT values (mean lifespans) for the fine root fraction of 0.36–1.19 years with a peak in the *Podocarpus* forest and a minimum in the *Ocotea* forest.

**Fig. 4:**
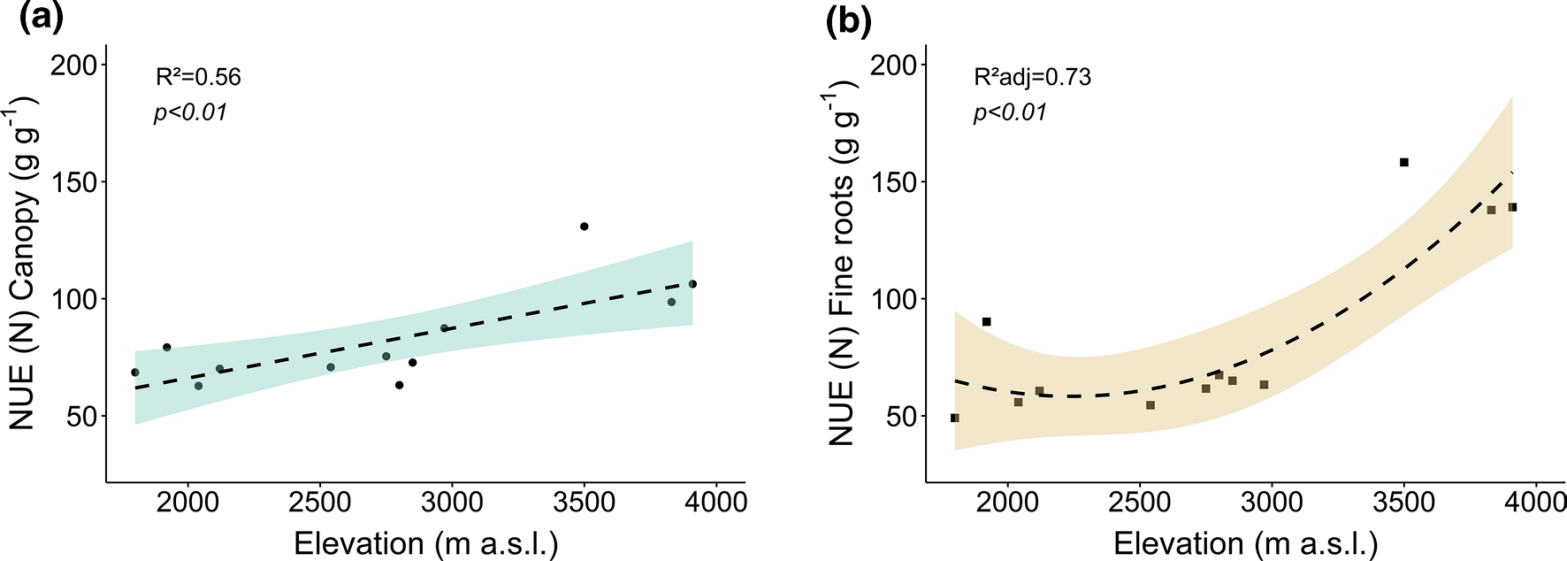
Change with elevation in the nitrogen use efficiency of **a** canopy productivity and **b** fine root productivity in the four forest types. Dashed lines indicate linear regressions or 2nd-order polynomial regressions (*n* = 12), colored areas the 95% confidence interval

Nitrogen use efficiency of productivity (NUE) was relatively similar for aboveground litter and fine root productivity in a given forest type (Table 2). Both efficiencies remained on a similar level in all forest types except in the *Erica forest*, which showed 50 to 120% higher values (Fig. 4). NUE reached its highest value in the fine root productivity of the *Erica* forest (145 g g^−1^). The NUE values were negatively related to NPP across the four forest types (Table S4).

### Carbon and nitrogen transfers to the soil via leaf and root litter

The leaf- and root-derived carbon and nitrogen fluxes were of similar size in the lower to upper montane forests, while the root litter flux greatly exceeded the leaf-born flux in the *Erica* forest (195.1 ± 9.7 g C m^−2^ year^−1^ and 2.8 ± 0.3 g N m^−2^ year^−1^ from fine roots vs. 0.5 ± 0.2 g C m^−2^ year^−1^ and 0.01 ± 0.005 g N m^−2^ year^−1^ from leaves) (Table 4).

**Table 4:** Estimated annual transfers of carbon and nitrogen to the soil with leaf litter production and with fine root mortality (sequential coring approach) in the four forest types

Ecosystem	C flux (g m^−2^ year^−1^)		N flux (g m^−2^ year^−1^)		N (mg g^−1^)		C: N ratio (mg mg^−1^)	
	Fine root litter	Leaf litter	Fine root litter	Leaf litter	Fine root litter	Leaf litter	Fine root litter	Leaf litter
Lower montane forest	211.72 ± 7.44 a	298.48 ± 16.23 b	7.67 ± 1.32 a	8.53 ± 0.97 a	16.47 ± 2.78 a	13.87 ± 1.21 a	29.22 ± 4.71 a	37.39 ± 2.86 a
*Ocotea* forest	178.91 ± 19.76 a	163.88 ± 16.97 a	6.42 ± 0.35 a	4.04 ± 0.61 b	17.02 ± 0.66 a	12.37 ± 0.62 b	27.71 ± 1.54 a	45.26 ± 1.89 b
*Podocarpus* forest	171.26 ± 27.79 a	160.56 ± 16.39 a	5.51 ± 0.92 a	3.84 ± 0.93 a	15.35 ± 0.27 a	11.84 ± 1.68 a	31.16 ± 0.40 a	46.25 ± 6.51 a
*Erica* forest	195.13 ± 9.65 a	0.50 ± 0.15 b	2.83 ± 0.25 a	0.011 ± 0.005 b	6.92 ± 0.30 a	12.80 ± 0.11* b	69.48 ± 3.25 a	48.73 ± 5.24 b

Also given are the N content and the C:N ratio in the litter fractions. Assuming a steady state of fine root production and mortality, fine root production is equal to fine root litter production. Given are means ± SE (*n* = 3). Different lower case letters indicate significant differences between fine root and leaf litter fluxes in a given ecosystem according to ANOVA (*P* < 0.05)

## Discussion

### Decreasing net primary productivity with elevation and its causes

Our replicated study of key productivity components in four mountain forest types along a 2000-m elevation gradient in tropical East Africa shows a continuous decline of total NPP with increasing elevation, that was associated with a marked belowground shift in carbon allocation toward the fine root system. With a decrease in mean annual temperature from 14.7 to 9.3 °C, total NPP and its aboveground components NPP-aboveground litterfall and NPP-aboveground wood decreased by roughly 30% from the lower montane forest to the montane *Podocarpus* forest, contradicting the conclusions of Luyssaert et al. (2007) and Michaletz et al. (2014) who reported minor or no temperature effect on forest productivity in warm-temperate to tropical climates with MAT > 10 °C.

Total NPP values recorded in the four montane forest types on Mt. Kilimanjaro (2.8–8.5 Mg C ha^−1^ year^−1^) were in the lower range of data from pantropical surveys of forest productivity (3.1–21.7 Mg C ha^−1^ year^−1^) (e.g. Clark et al. 2001b; Kitayama and Aiba 2002; Aragão et al. 2009), but similar to figures from tropical montane forests in Rwanda (9.2 ± 2.1 Mg C ha^−1^ year^−1^) (Nyirambangutse et al. 2017) and the Ecuadorian and Peruvian Andes (3.9–6.4 and 4.1–7.1 Mg C ha^−1^ year^−1^, respectively; Moser et al. 2011; Girardin et al. 2010). The subalpine *Erica* forest at the tree line (2.8 Mg C ha^−1^ year^−1^) had a 30% lower NPP than the corresponding upper montane forest at high elevations in the Ecuadorian Andes (3060 m a.s.l.) (4.1 Mg C ha^−1^ year^−1^) (Moser et al. 2011) and almost 65% lower NPP than the tree line forest in the Peruvian Andes (3500 m a.s.l.) (7.9 Mg C ha^−1^ year^−1^) (Malhi et al. 2017). Several factors may have contributed to this difference, among them the local species composition and related differences in stand structure, but also the regional climatic and edaphic conditions. The Ecuadorian upper montane forest has a larger stem density, basal area and mean tree height than the *Erica* forest in East Africa. The total NPP estimates in the Peruvian forest include measurements of small tree (2–10 cm DBH) productivity, leaf herbivory and carbohydrate transfer to the mycorrhiza (Malhi et al. 2017), which have not been quantified in our study. However, even when we increase our NPP value by 20% to account for these NPP components, the Peruvian tree line forest is still more productive than its counterpart in eastern Africa. Both Andean upper montane forests grow under higher precipitation and temperature levels than the *Erica* forest on Mt. Kilimanjaro (Moser et al. 2011; Malhi et al. 2017), demonstrating the transition to a high-elevation climate with relatively low precipitation on top of the volcano (1050 mm year^−1^), whereas the uplift of moist air masses proceeds to higher elevations in the Andes.

Until recently, the change in forest productivity with elevation in tropical mountains was only studied with respect to aboveground NPP, while ignoring root productivity (Raich et al. 1997; Kitayama and Aiba 2002; Wang et al. 2003). Only the two studies from Peru and Ecuador by Girardin et al. (2010) and Moser et al. (2011) have investigated root production as well, allowing conclusions on elevational change in total NPP. The observed NPP trends differ between the three transects. While total NPP decreased more or less continuously from the lower montane to the upper montane forest in the Ecuadorian Andes (Moser et al. 2011; Leuschner et al. 2013), an abrupt productivity decline from the submontane (1500 m) to the cloud forest (1700 m) and nearly constant productivity higher upslope was found in the Peruvian Andes (Girardin et al. 2010; Malhi et al. 2017). In contrast, we found on Mt. Kilimanjaro a gradual NPP decline from the lower montane to the upper montane (*Podocarpus*) forest and an abrupt decrease toward the subalpine *Erica* forest. Carbon residence time in the wood increased from the more productive lower montane forest to the upper montane *Podocarpus* forest and sharply decreased to the *Erica* forest, revealing a hump-shaped curve along the slope. This pattern may partly be explained by the change from a forest with evergreen broadleaf trees to a conifer-dominated forest with *Podocarpus latifolia* and finally to an *Erica trimera* forest, which is exposed to more extreme minimum temperatures and of lower stature than the forests further downslope.

The data from Tanzania and Ecuador suggest that a major driver of the NPP decline is the reduction in leaf area index (LAI). In both transects, LAI decreased by more than 50% over a 2000-m elevation gradient from lower montane to upper montane elevation (Leuschner et al. 2007; Rutten et al. 2015). A decreasing photosynthetic capacity (A_sat_) with elevation may also contribute to the productivity decline. According to a pan-tropical A_sat_ database, the light-saturated net photosynthesis of trees at ambient temperature decreases on average by 1.3 µmol CO_2_ m^−2^ s^−1^ per 1 km altitude increase in tropical mountains (Wittich et al. 2012). The decrease is typically linked to an altitudinal decrease in mass-related foliar N content (Moser et al. 2011; Schellenberger Costa et al. 2017). The results from the elevation transect in the Andes and eastern Africa indicate that both leaf area and photosynthetic capacity are influenced by the N supply along the mountain slope with the consequence that local and regional differences in soil fertility may cause somewhat different elevational trends in LAI, A_sat_ and total NPP.

Soil chemical and biological analyses in the Tanzanian and Ecuadorian transects indicate that N availability decreases with elevation in tropical mountains. On Mt. Kilimanjaro, both soil and leaf C:N ratio increased with elevation toward the subalpine *Erica* forest, while fine root N content decreased, in conjunction with declining decomposition rate (Becker and Kuzyakov 2018). Moreover, we found a large increase in the N use efficiency of canopy and root productivity from the montane *Podocarpus* forest to the higher *Erica* forest on Mt. Kilimanjaro, linked to a pronounced shift in C allocation toward the root system. These findings explain the significant negative relation that exists between total NPP and soil C:N ratio in our four studied forest types. The elevational temperature decrease can influence N cycling through a negative impact on various processes, among them ammonification and nitrification, the diffusion of inorganic N compounds in the soil, the activity of mycorrhiza, and root N uptake (Chapin 1980; Pendall et al. 2004; Marschner 2012; Robertson and Groffman 2015). We assume that the temperature effect on N cycling and N supply and resulting reductions in leaf area (and possibly photosynthetic capacity) represent a major pathway through which elevation is acting on total C gain. In addition, tree growth was found to be stimulated by moderate nitrogen fertilization at the tree line ecotone in the Swiss Alps (Möhl et al. 2018), supporting our assumption of nutrient limitation of aboveground tree productivity at high elevations. The replacement of macrophyllous, broadleaved trees by southern hemispheric conifers at the upper montane zone and ericoid trees with more sclerophyllous leaves and their specialized ericoid mycorrhiza at the subalpine zone can also be interpreted as an indication of increasing nutrient limitation at higher elevations. There is a feedback between environmental conditions and functional composition. Of course, the species turnover with increasing elevation has an impact on forest productivity. It has been shown that the set of functional traits associated with a plant community is a strong driver of spatial variation in forest productivity (Fyllas et al. 2017). The plant species along the slope reflect in their traits the environmental change with elevation, which results from adaptation and acclimation to the local conditions  (Chapin et al. 2011). We can see these adaptations along the slope of Mt. Kilimanjaro with the substitution of fast-growing species with high SLA as *Macaranga kilimandscharica* in the lower montane forest, where light is limiting, by slow-growing species as *Podocarpus latifolius* with lower SLA and nodules with mycorrhiza in the upper montane forest, with stronger N limiting conditions. Additionally, at the tree line, the sclerophyllous leaves and ericoid mycorrhiza, lower height and biomass of the monodominant *Erica trimera* are an adaptation to the extreme conditions (e.g. N limitation, strong wind, high irradiance, low temperature) at high elevations. Therefore, we focus our discussion on the change from light limiting conditions to N shortage along the slope, which result in lower LAI, lower photosynthetic capacity and thus, a decrease of total NPP. At the same time, the increasingly N limited conditions with elevation trigger a shift of the C allocation from aboveground to belowground organs in the woody plants.

Clearly, other environmental factors besides N supply must be responsible for the elevational NPP decrease as well, notably thermal limitation of meristem activity (Körner 2012). However, there is no physiological reason to assume that low temperatures should constrain aboveground meristematic activity more than belowground meristematic activity. Thus, a direct thermal effect on meristematic activity cannot explain the pronounced allocation shift toward root growth. Another factor possibly limiting forest productivity at higher elevations is reduced irradiance due to persistent cloud cover at montane elevation, as it was assumed for the Peruvian transect (Malhi et al. 2017). However, on Mt. Kilimanjaro, cloudiness was highest in the *Ocotea* forest belt (Hemp 2006), where total NPP was still comparatively high with 6.4 Mg C ha^−1^ year^−1^, exceeding the NPP of the *Erica* forest higher up more than twofold. This makes it unlikely that enhanced light limitation in the cloud belt plays an important role in this transect.

The regression coefficients suggest that precipitation (MAP) is influencing NPP, but to a lesser degree than temperature and related phenomena such as nutrient supply. Our results are in line with an analysis of large NPP data sets that revealed an increase in the NPP of tropical forests with MAP until fairly high values (˃ 2500 mm), followed by a decrease at even higher rainfall possibly due to reduced irradiance or reduced soil biological activity and N mineralization in wet soil (Schuur 2003; Del Grosso 2008). On Mt. Kilimanjaro MAP (not exceeding 2200 mm) seems to promote forest NPP across the entire sequence of forest belts present on this mountain.

### Fine root productivity and elevational shifts in carbon allocation

By including fine root production, our study is among the few that allows estimating the total NPP of tropical forest along elevation gradients and analyzing shifts in carbon allocation. These productivity data have to be interpreted with caution, however. The sequential coring and ingrowth core methods yielded largely different results, as has been found in other studies (Vogt et al. 1998; Hertel and Leuschner 2002; Moser et al. 2010). Both approaches have their shortcomings. The sequential coring technique with the minimum–maximum calculation approach is believed to underestimate fine root productivity when the seasonal variation in total fine root mass (live and dead) is larger than the difference between highest and lowest fine root mass count, and fine root decomposition is rapid (Vogt et al. 1998). The ingrowth core approach investigates root growth under more artificial conditions and starts from an injured root system. The growth rates estimated by this method may give an impression of the root system’s regeneration potential rather than reflecting root productivity under natural conditions. As in the study of Moser et al. (2010), our ingrowth core productivity values were several times smaller than those obtained from sequential coring, especially at high elevations. Based on the ingrowth core data, we calculated fine root productivities in the range of 0.2–0.7 Mg C ha^−1^ year^−1^ and a mean carbon residence time between 1.4 and 5.0 years for the fine root fraction in the four forest types (Table S2). This is higher than fine root longevities derived from mini-rhizotron observation in tropical montane forests in the Ecuadorian Andes (0.70–0.95 years; Graefe et al. 2008) and also exceeds the average fine root longevity of 1.32 years found by Gill and Jackson (2000) for tropical forests in a meta-analysis (mostly sequential coring studies). According to our sequential coring data, average fine root longevity was 0.4–1.2 years, which matches these figures better. We thus suggest that the sequential coring data may give a more realistic picture of fine root dynamics than the ingrowth core results, which have greater value for site comparison.

Three main conclusions may be drawn from our fine root productivity (sequential coring) data: (1) Fine roots are the most dynamic component in the studied tropical mountain forests, exceeding even the leaf fraction. (2) With 26 to 72% of total NPP, fine root productivity consumes more carbon than wood production. (3) In contrast to the other productivity components, fine root growth seems to be controlled mostly by intrinsic factors (probably sink strength and carbohydrate availability), as significant relations to climatic, edaphic and stand structural parameters were lacking. With the resource balance hypothesis (Bloom et al. 1985), the pronounced shift in carbon allocation from leaf growth (NPP-aboveground litterfall) to fine root growth from the upper montane *Podocarpus* to the subalpine *Erica* forest is interpreted as a change from predominant light limitation to nutrient (or water) limitation of growth. In our study, decreasing N supply with elevation is a plausible explanation, but a deficiency in other elements such as phosphorus (P) or potassium is possible at tropical high-elevation sites (Graefe et al. 2010). Yet, leaf mass N:P ratios suggest primarily N limitation of growth on Mt. Kilimanjaro (Townsend et al. 2007). A belowground shift of C allocation has been concluded from studies on productivity or biomass changes along elevation transects in other tropical mountains (Leuschner et al. 2007, 2013; Girardin et al. 2010; Moser et al. 2011) and temperate mountains (Hertel and Schöling 2011; Mao et al. 2015) as well. Fine root studies at the alpine and arctic tree line also indicate that the C allocation preference of trees shifts belowground in cold environments (Ruess et al. 2003; Kubisch et al. 2017). As these studies were conducted mostly in humid climates, it is unlikely that water limitation is a possible driver of the allocation shift. Even though Mt. Kilimanjaro receives relatively low precipitation on its top, the amount in the *Erica* forest belt (1050 mm) seems to be high enough to exclude a main role of water shortage.

Thus, we assume that high-elevation forests in tropical mountains are not only limited by low temperatures that restrict meristem activity, but their growth seems to be constrained by nutrient (in particular N) shortage as well. Whether reductions in N supply, N diffusion in the soil, or impaired N uptake and relocation in the plant are primarily limiting, has to be addressed in physiological and soil chemical studies that should include soil warming experiments.

Fine roots are not only large sinks for photosynthates but also important sources of organic C and N which is transferred to the soil upon root death and via root exudation and transfer to the mycorrhiza (Rasse et al. 2005; Godbold et al. 2006). Our fine root productivity data suggest that the C and N flux to the soil with root litter is roughly of similar importance as the leaf litter flux in the lower montane and *Ocotea* forest, while it represents an important C and N source supplied by woody plants in the *Erica* forest. Comparable results have been obtained from Ecuadorian forests at high elevations (Röderstein et al. 2005). This highlights the need to include fine root dynamics in biogeochemical studies in forests of the tropics and elsewhere (Litton et al. 2007).

## Conclusions

To our knowledge, this is the first study on forest productivity and its main above- and belowground components along an elevation transect on a paleotropical mountain. In accordance with earlier studies in neotropical elevation transects, we found a more or less continuous decrease of total NPP with elevation, confirming a pronounced temperature influence on forest productivity also in the temperature interval between 10 and 15 °C. Comparison with other tropical and non-tropical elevation transect studies reveals that the pronounced belowground shift in carbon allocation with elevation is a characteristic feature of mountain forests worldwide, suggesting increasing growth impairment through N (nutrient) limitation toward higher elevations, which seems to act independently from direct low-temperature effects on meristem activity. Inherent bias in the fine root productivity data warrants caution in the interpretation of the calculated fine root production data. Nevertheless, from the remarkable constancy of root productivity along the slope and the fairly good agreement with earlier neotropical fine root studies, we assume that the observed patterns are mostly valid. Future direct observational studies with mini-rhizotrons could provide a welcome independent estimate of fine root productivity. We conclude that initiatives to measure all major components of forest productivity along elevation gradients can generate valuable additional insights into the climate dependence of carbon cycling in woodlands.

## Supplementary Information

Below is the link to the electronic supplementary material.

Supplementary file1 (DOCX 100 KB)

## Data Availability

The datasets analyzed during the current study are available from the corresponding author on reasonable request.
