# Supplementary material for: Climate implications on forest above- and belowground carbon allocation patterns along a tropical elevation gradient on Mt. Kilimanjaro (Tanzania)
Source: Oecologia. 2021 Feb 25;195(3):797–812. doi: 10.1007/s00442-021-04860-8 (PMC7940314; doi:10.1007/s00442-021-04860-8)
Supplement: Supplementary file 1 — Supplementary file1 (DOCX 100 KB) [file 442_2021_4860_MOESM1_ESM.docx]

**Supplementary material**

**Table S1.** Carbon and nitrogen content in fine root biomass of four forest ecosystems along the elevation on Mt. Kilimanjaro (Sierra Cornejo et al. 2020). Given are means ± SE (n=3).

| Ecosystem | Content in fine roots (mg g¯¹) | |
| --- | --- | --- |
|  | Carbon | Nitrogen |
| Lower montane forest | 455.2 ± 14.2 | 16.5 ± 2.8 |
| *Ocotea* forest | 470.3 ± 17.3 | 17.0 ± 0.7 |
| *Podocarpus* forest | 478.0 ± 4.8 | 15.4 ± 0.3 |
| *Erica* forest | 479.0 ± 2.3 | 6.9 ± 0.3 |

**Table S2.** Net Primary Productivity (NPP), NPP allocation to the components, carbon residence time (CRT), carbon (C) and Nitrogen (N) fluxes from fine roots to the soil via root dead; and fine root:leaf litter ratio of four forest ecosystems along the elevation on Mt. Kilimanjaro calculated using the ingrowth core method for fine root NPP estimation. Given are means ± SE (n=3). Values in parentheses give carbon fluxes (in Mg C ha^-1^ yr^-1^). aLF – aboveground litter fall; aW – aboveground wood; FR – fine roots; CR – coarse roots.

C content of fine roots obtained by the ingrowth core approach was determined using a C/N elemental analyzer (Vario EL III, Hanau, Germany). Three samples per plot were analyzed, with each sample consisting of two root subsamples that were mixed.

**Table S3.** Pearson correlation analysis between elevation, climatic, edaphic and stand structural variables for the four ecosystems along the elevation. Pearson correlation coefficient *r* and *P* are given. Elevation in m a.s.l., mean annual temperature (MAT) in °C, mean annual precipitation (MAP) in mm, aboveground biomass (AGB) in Mg ha^-1^, and basal area in m² ha^-1^.

Climatic data: MAT from Appelhans et al. (2015) and MAP from Hemp (2006), topographic and stand structure data from Hemp (unpublished data), *Erica* forest stand structural data from Schellenberger Costa (unpublished data), soil data from Becker (unpublished data). Values in bold indicate significant correlation (*p*< 0.05). Positive correlations are marked with (+) and negative ones with (-).

**Table S4.** Relation of NUE from aboveground litter fall and fine roots to elevation. NPP total in Mg ha^-1^ yr^-1^ and NUE in g g^-1^.

|  |  | NUE Canopy | |  |  | NUE Fine roots | |
| --- | --- | --- | --- | --- | --- | --- | --- |
|  |  | *r²adj* | *P* |  |  | *r²adj* | *P* |
| NPP total | **-** | **0.65** | **<0.01** |  | **-** | **0.70** | **<0.001** |

Significant relations are marked in bold *(P<0.05)* and negative relations are indicated by (-).


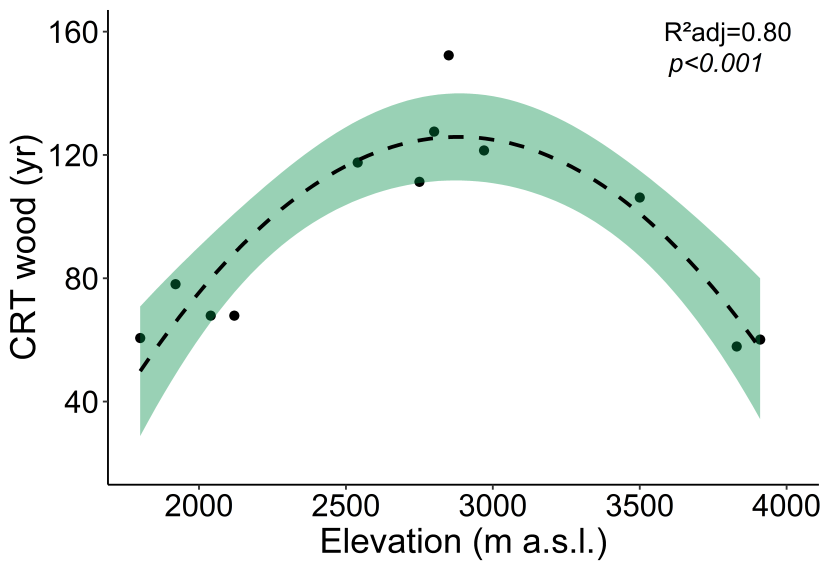


**Fig. S1.** Change with elevation in the carbon residence time (CRT) of aboveground wood in the four forest types. The dashed line indicates a 2^nd^-order polynomial regression (n= 12), the colored area the 95 % confidence interval.
